# Investigating the relationship between self-rated health and social capital in South Africa: a multilevel panel data analysis

**DOI:** 10.1186/s12889-015-1601-0

**Published:** 2015-03-19

**Authors:** Yan Kwan Lau, John E Ataguba

**Affiliations:** Health Economics Unit, School of Public Health and Family Medicine, University of Cape Town, Observatory, 7925 Cape Town, South Africa

**Keywords:** Social capital, Self-rated health, South Africa

## Abstract

**Background:**

The relationship between social capital and self-rated health has been documented in many developed compared to developing countries. Because social capital and health play important roles in development, it may be valuable to study their relationship in the context of a developing country with poorer health status. Further, the role of social capital research for health policy has not received much attention. This paper therefore examines the relationship between social capital and health in South Africa, a country with the history of colonialism and apartheid that has contributed to the social disintegration and destruction of social capital.

**Methods:**

This study uses data from the National Income Dynamics Study (NIDS), the first nationally representative panel study in South Africa. Two waves of the NIDS were used in this paper – Wave 1 (2008) and Wave 2 (2010). Self-rated health, social capital (individual- and contextual-level), and other covariates related to the social determinants of health (SDH) were obtained from the NIDS. Individual-level social capital included group participation, personalised trust and generalised trust while contextual-level or neighbourhood-level social capital was obtained by aggregating from the individual-level and household-level social capital variables to the neighbourhood. Mixed effects models were fitted to predict self-rated health in Wave 2, using lagged covariates (from Wave 1).

**Results:**

Individual personalised trust, individual community service group membership and neighbourhood personalised trust were beneficial to self-rated health. Reciprocity, associational activity and other types of group memberships were not found to be significantly associated with self-rated health in South Africa. Results indicate that both individual- and contextual-level social capital are associated with self-rated health.

**Conclusion:**

Policy makers may want to consider policies that impact socioeconomic conditions as well as social capital. Some of these policies are linked to the SDH. We contend that the significant social capital including community service membership can be encouraged through policy in a way that is in line with the values of the people. This is likely to impact on health and quality of life generally and lead to a reduction in the burden of disease in South Africa considering the historic context of the country.

## Background

There has been a prominent increase in research investigating the role that social capital plays in health outcomes. Much of the research has focused on developed countries [1-6]. Given that both social capital and health have demonstrated to play important roles in development [7], it may be valuable to study their relationship in the context of a developing country. Although social capital is an ‘essentially contested’ concept like race, class and gender [8], some authors distinguish three basic types including bonding, bridging and linking social capital. Bonding social capital relates to relations that occur within-group as members of the group are fairly homogenous (e.g. race, gender, occupation, socio-economic status), while bridging social capital concerns with between-group relations and the individuals involved are more dissimilar to each other. Linking social capital, on the other hand, is similar to bridging social capital but takes into account power differentials that are present in some relations or networks [8]. In public health research, the most cited definitions of social capital are from the works of Bourdieu, Coleman and Putnam (see [9]). Briefly, Bourdieu P [10] considers social capital as resources such as money, status, and information – actual or potential – that are linked to a network. Coleman JS [11] conceptualises social capital according to its function to facilitate certain actions that would have otherwise been impossible. Putnam RD [12] refers to social capital as “features of social organization such as networks, norms and social trust that facilitate coordination and cooperation for mutual benefit (p. 2).”

Albeit social capital is recognised to be related in some ways to health and development [7], the role of social capital research for health policy has not received much attention. Social capital, a form of capital, is a facilitating factor for the use of health services in Africa [13]. As argued elsewhere, social capital can form a basis for empowering citizens to “participate equally in the networks that generate social capital” and it could be used to devolve governance to local communities wherever feasible ([14] p.16). Similarly, to build social capital, the government could use specific policies that target housing conditions, income security, community environmental improvements, recreational facilities, leisure time enhancement, and educational interventions [14]. These areas form the basic social determinants of health.

In South Africa, the history of colonialism and apartheid has contributed to the social disintegration and destruction of social capital of the country, particularly, within black communities [15,16]. Since the end of apartheid and the transition to democracy in 1994, South Africa’s policies have focused on the importance of social capital and the beneficial role it plays towards a cohesive society and the well-being of its people [17]. Further, results from systematic reviews indicate that the relationship between social capital and health is more consistent in contexts where there is more income inequality [18]. The World Bank’s most recent estimate of South Africa’s Gini coefficient of income inequality stands at 63.1 – one of the highest in the world [19]. Therefore, in addition to other well-known social determinants of health, it is worthwhile to examine the relationship between social capital and health in South Africa.

However, there has been limited research investigating the multifaceted relationship between social capital and various health outcomes in South Africa generally. Only a few studies have investigated aspects of this relationship. Some did not use nationally representative samples [20-23] while some used the National Income Dynamics Study (NIDS) panel dataset [24,25] but in a cross-sectional manner.

Given the paucity of datasets in many African countries, available data in South Africa provide an avenue to examine the relationship between social capital and health, especially given its own historic context. Specifically, the paper used two waves of NIDS data (2008 and 2010) in a multi-level framework to examine the prospective relationship between social capital and self-rated health, while controlling for socioeconomic variables. Thus, social capital as used in this paper lends itself best to Uphoff’s [26] dimensions of social capital: structural and cognitive social capital. The structural dimension is directly observable and refers to forms of social organisation and networks that contribute to cooperation; the cognitive dimension refers to mental processes that promote social cooperation such as trust [26]. These dimensions can also be applied to Putnam’s definition of social capital, which was the intended conceptualisation for the NIDS [17]. In this paper, the cognitive component of social capital comprises personalised trust, generalised trust, reciprocity and associational activity; and group membership forms the structural domain. This paper hypothesises that even after controlling for other social determinants of health (e.g. educational attainment, employment status and household income), social capital will be associated with self-rated health in South Africa.

## Methods

### Data

The data came from the National Income Dynamics Study (NIDS) conducted biennially by the Southern Africa Labour and Development Research Unit (SALDRU) based at the University of Cape Town. This is the first nationally representative panel study of households in South Africa. Full details of NIDS are available elsewhere [27]. Briefly, NIDS used a stratified, two-stage cluster sample design to sample households in the nine provinces of South Africa. First, 400 primary sampling units (PSUs) were chosen from a master sample of 3000 PSUs identified by Statistics South Africa in 2003. Subsequently, the chosen PSUs were randomly sampled within each stratum of 53 district councils, which were also proportional to the master sample’s allocation of PSUs in each stratum. PSUs are derived from Census Enumeration Areas and served as the unit of “neighbourhood” in this paper. Waves 1 and 2 were conducted in 2008 and 2010 respectively. About 7300 households (i.e. 16878 individuals) were interviewed at the end of Wave 1 (in 2008), corresponding to a response rate of 69% [27]. The overall attrition rate between Waves 1 and 2 was about 22% [28]. Procedures taken to track respondents and to ensure data consistency between the two waves are detailed elsewhere [28]. This paper used data collected through the adult questionnaires (administered to every household member over 15 years old) in Waves 1 and 2, and also the household questionnaire from Wave 1. Further, it only included subjects whose responses for the outcome variable of interest, self-rated health, were recorded in both 2008 and 2010 (n = 12,093). The final sample size used for analysis was 8866 due to missing data in the explanatory variables. A simple sample selection test compares the means and proportions of key variables between the full sample and the sub-sample used in the analysis reveals the absence of any substantial sample selection issue.

### Outcome variable

The main outcome of interest was individual self-rated health in Wave 2. Respondents were asked the following: “How would you describe your health at present? Would you say it is excellent, very good, good, fair or poor?” Self-rated health was dichotomised to “poor health” = 1 (fair or poor) and “good health” = 0 (excellent, very good or good), as has been done in previous studies [29-31]. Moreover, a reliability test was also performed to confirm the dichotomisation [32]. Self-rated health has been well-established as a reliable predictor of mortality in a variety of contexts [33,34], including the South African context [35].

### Social capital variables

Individual-level social capital contained in the NIDS included group participation, personalised trust and generalised trust. Respondents were asked to indicate their membership in various groups. Group membership was then categorised according to the groups’ functions: financial (stokvel^a^ and burial society) [36], production (farmer’s association, informal trader’s group, community garden group, sewing group), community service (school committee, water committee, development committee, youth groups, women’s association, men’s association), political/union (tribal authority and trade union), and private interest (singing/music group, study group, sports group) [37,38]. Personalised trust was assessed by asking respondents, “Imagine you lost a wallet or purse that contained R200^b^ and it was found by someone who lives close by. Is it very likely or not likely at all to be returned with the money in it?” Generalised trust asked: “Imagine you lost a wallet or purse that contained R200 and it was found by a complete stranger. Is it very likely or not likely at all to be returned with the money in it?” These two items of trust were rated on a 3-point Likert scale: not likely at all, somewhat likely, and very likely. “Not likely” was operationalised as no trust (trust = 0), whereas the latter two as “has trust” (trust = 1). Reciprocity and associational activity was determined at the household-level where the former was assessed by the question “How common is it that neighbours help each other out?” and the latter by, “How common is it that neighbours do things together?” Both were rated on a 5-point Likert scale: never happens, rarely happens, not common, fairly common and very common. These categories were dichotomised into “high” (fairly common and very common) and “low.” Neighbourhood-level social capital was obtained by aggregating from the individual-level social capital and household-level social capital variables (binary variables) to the neighbourhood (i.e. Census Enumeration Areas).

### Covariates

Several individual-, household- and neighbourhood-level covariates from Wave 1 were considered (see Table 1). On the individual level: age, sex, race, marital status, education, employment status, self- rated health, urban, obese, smoking and number of household members. Household-level variables include household size and per capita household income quintiles. Lastly, a neighbourhood living environment deprivation index is included as a neighbourhood-level covariate. The index was derived by principal component analysis [39] using items from the South African Index of Multiple Deprivation’s “Living Environment Deprivation” domain [40] and the Service Deprivation Index [41]. This included households without piped water on site/in dwelling/borehole, without flush toilet on site/pit latrine with ventilation pipe, without electricity from main/generator, in informal dwelling/shack and unavailability of refuse removal at least once a week. The first principal component derived yields an eigenvalue of 2.90 and explains 58% of the total variation.

**Table 1:** **Demographic and health statistics in 2008**

**Variable**	**Category**	**n (%)**
*Individual-level variable*		
Gender	Male	3329 (37.55)
	Female	5537 (62.45)
Age	15-21	2017 (22.75)
	22-35	2306 (26.01)
	36-59	3359 (37.89)
	60+	1184 (13.35)
Self-rated health in Wave 1	Poor health	1891 (21.33)
	Good health	6975 (78.67)
Race	White	276 (3.11)
	Black	7346 (82.66)
	Coloured	1145 (12.91)
	Indian/Asian	99 (1.11)
Marital status	Married/Living with partner	3228 (36.41)
	Widow/Divorced/Separated/Never married	5638 (63.69)
Education	No schooling to some primary school	2748 (30.99)
	Completed primary school and some high school	4436 (50.03)
	Completed high school or more	1682 (18.97)
Employment status	Employed	1965 (22.16)
	Unemployed	6901 (77.84)
Urban/rural classification	Urban formal/Urban informal	3919 (44.20)
	Rural formal/Former tribal area	4947 (55.80)
Obese	Yes	2230 (25.15)
	No	6636 (74.85)
Smoker	Yes	1642 (18.52)
	No	7224 (81.48)
*Household-level variable*		
Household size [mean (SD)]	(continuous)	5.28 (3.18)
Per capita household income quintiles	5^th^ quintile	1021 (11.52)
	1^st^ quintile	1762 (19.87)
	2^nd^ quintile	2123 (23.95)
	3^rd^ quintile	2046 (23.08)
	4^th^ quintile	1914 (21.59)
*Neighbourhood-level variable*		
Living environment index deprivation index [mean (SD)]	(continuous)	0.34 (1.68)

Note: Reference categories used for multilevel models for each variable are listed first and bolded.

### Analytical methods

A multilevel analysis was conducted to account for the hierarchical nature of the data such that individuals (level 1) are nested within households (level 2), which are in turn, within neighbourhoods (level 3). To begin, indirect standardisation of the dichotomous outcome of self-rated health by age and sex was applied. This attempts to correct the distribution of self-rated health by comparing it with that expected of the actual age/sex distribution [42]. Thereafter, eight mixed-effects linear models were fitted: Model 0 is the null model; Model 1 consists of all covariates without any social capital indicators; Models 2 to 6 build on Model 1, separately adding on personalised trust, generalised trust, norms of reciprocity, norms of association, and various types of group memberships respectively; lastly, Model 7 is the full model and includes all variables. The software Stata 11.2 [43] was used to carry out all analyses.

### Ethical approval

Ethical approval for this study was obtained from the Human Research Ethics Committee of the University of Cape Town.

## Results

The majority of the sample (78.67%) reported good health in Wave 1 (Table 1). 82.66% were black and 37.55% were male while 36.41% indicated they were married or living with their partners. Further, over 80% of the sample did not complete high school, and just under 80% reported to be unemployed. More than half lived in a rural area and had an average of 5 members per household (Table 1). About a quarter of the sample was classified as obese (with body mass index (BMI) > 30) and just under a fifth reported to be current smokers in 2008 (Table 1). With regards to social capital indicators (Table 2), there were low levels of trust: only 23.27% reported having personalised trust, and it was lower at 12.01% for generalised trust. Just over a half of the sample reported being a member of any group, with the largest participation in financial groups. Lastly, norms of reciprocity and association recorded at household levels were a lot higher when compared to either trust indicators.

**Table 2:** **Descriptive statistics of social capital indicators in wave 1**

**Variable**	**n (%)**
*Individual-level indicator*	
Personalised trust	2063 (23.27)
Generalised trust	1065 (12.01)
Group membership	
Financial group	2085 (23.52)
Production group	155 (1.75)
Private interest group	1051 (11.85)
Community service group	733 (8.27)
Political group	614 (6.93)
*Household-level indicator*	
Norms of reciprocity (high)	5850 (65.98)
Norms of association (high)	5399 (60.90)
*Neighbourhood-level indicator*	mean (SD)
Proportion of neighbourhood personalised trust (high)	0.24 (0.20)
Proportion of neighbourhood generalised trust (high)	0.12 (0.13)
Proportion of neighbourhood reciprocity (high)	0.66 (0.20)
Proportion of neighbourhood associational activity (high)	0.61 (0.22)
Proportion with financial group membership	0.24 (0.16)
Proportion with production group membership	0.02 (0.03)
Proportion with community service group membership	0.08 (0.09)
Proportion with private interest group membership	0.11 (0.09)
Proportion with political group membership	0.07 (0.10)

The results for the three-level mixed-effects linear regression models of poor health in 2010 on predictors from 2008 are presented in Table 3. Compared to the null model, Model 1 indicated that reported poor health in Wave 1 was a significant predictor of poor health in Wave 2 (p ≤ 0.01). Being black (compared to white) was significantly associated with poor health (p ≤ 0.05), but this was not significant for the other race groups. Belonging to household income quintiles 1 to 4 (compared to the 5th quintile) were positively associated with poor health (all p ≤ 0.05). On the other hand, being married, completed primary school or more, being employed, and household size, were negatively associated with poor health (p ≤ 0.01 for all, except married/living with partner where p ≤ 0.05). All these associations were similar in Model 2 when individual- and neighbourhood-level personalised trust were added except for being black, which was no longer significantly related to poor health. Both individual-level and neighbourhood-level personalised trust were negatively associated with poor health (both p < 0.01). In Model 3, all associations from Model 1 were retained, and only individual-level generalised trust was found to be significantly negatively associated with poor health (p ≤ 0.01), but not at the neighbourhood-level (p > 0.10). Models 4 and 5 showed that neither norms of reciprocity nor association were significantly associated with poor health on any level (all p > 0.10); associations with other covariates were the same as in Model 1. In Model 6, only individual membership in community service groups (p ≤ 0.01) and neighbourhood-level membership in a financial group (p ≤ 0.05) were associated negatively with poor health; significant associations with all the other types of group memberships were not detected. In Model 7, which included the full set of variables, the social capital indicators that remained statistically significant were individual personalised trust (p ≤ 0.01), community service group membership (p ≤ 0.01), neighbourhood personalised trust (p ≤ 0.01), neighbourhood generalised trust (p ≤ 0.05) and neighbourhood membership in a financial group (p ≤ 0.05).

**Table 3:** **Results for three-level mixed-effects linear regression models of poor health in 2010 on predictors from 2008**

**Variable**	**Co-efficient (standard error)**							
	**Model 0**	**Model 1**	**Model 2**	**Model 3**	**Model 4**	**Model 5**	**Model 6**	**Model 7**
*Individual-level variables*								
Self-rated health in Wave 1	-	0.133***	0.132***	0.134***	0.133***	0.134***	0.133***	0.132***
		(0.009)	(0.009)	(0.009)	(0.009)	(0.009)	(0.009)	(0.009)
Race: Black	-	0.048**	0.028	0.049**	0.050**	0.050**	0.061**	0.029
		(0.025)	(0.025)	(0.025)	(0.025)	(0.025)	(0.026)	(0.027)
Coloured	-	0.028	−0.002	0.027	0.029	0.029	0.032	−0.002
		(0.026)	(0.027)	(0.026)	(0.026)	(0.026)	(0.027)	(0.027)
Indian/Asian	-	0.049	0.030	0.047	0.049	0.049	0.047	0.027
		(0.047)	(0.046)	(0.047)	(0.047)	(0.047)	(0.047)	(0.046)
Married/Living with partner	-	−0.01**	−0.014*	−0.015**	−0.015**	−0.015	−0.013*	−0.012
		(0.007)	(0.007)	(0.007)	(0.007)	(0.007)	(0.008)	(0.008)
Education: Completed primary and some high school	-	−0.040***	−0.040***	−0.041***	−0.041***	−0.041***	−0.040***	−0.037***
		(0.008)	(0.008)	(0.008)	(0.008)	(0.008)	(0.008)	(0.008)
Completed high school or more	-	−0.059***	−0.056***	−0.058***	−0.059***	−0.059***	−0.057***	−0.053***
		(0.011)	(0.011)	(0.011)	(0.011)	(0.011)	(0.011)	(0.011)
Employed	-	−0.027***	−0.028***	−0.028***	−0.028***	−0.027***	−0.029***	−0.029***
		(0.009)	(0.009)	(0.009)	(0.009)	(0.009)	(0.010)	(0.010)
Urban	-	0.024	0.023	0.024	0.022	0.023	0.021	0.020
		(0.016)	(0.015)	(0.016)	(0.016)	(0.016)	(0.016)	(0.016)
Obese	-	−0.002	−0.003	−0.002	−0.002	−0.002	−0.002	−0.002
		(0.008)	(0.008)	(0.008)	(0.008)	(0.008)	(0.008)	(0.008)
Smoker	-	0.017*	0.016*	0.017*	0.017*	0.017*	0.015*	0.014
		(0.009)	(0.009)	(0.009)	(0.009)	(0.009)	(0.009)	(0.009)
Personalised trust	-	-	−0.037***	-	-	-	-	−0.031***
			(0.009)					(0.010)
Generalised trust	-	-	-	−0.030	-	-	-	−0.016
				(0.011)***				(0.012)
Group membership								
Financial group	-	-	-	-	-	-	−0.002	−0.002
							(0.009)	(0.009)
Production group	-	-	-	-	-	-	−0.036	−0.037
							(0.026)	(0.026)
Private interest group	-	-	-	-	-	-	−0.005	−0.005
							(0.011)	(0.011)
Community service group	-	-	-	-	-	-	−0.039***	−0.038***
							(0.013)	(0.013)
Political group	-	-	-	-	-	-	0.004	0.003
							(0.016)	(0.016)
*Household-level variables*								
Household size	-	−0.004***	−0.004***	−0.004***	−0.004***	−0.004***	−0.003***	−0.004***
		(0.001)	(0.001)	(0.001)	(0.001)	(0.001)	(0.001)	(0.001)
1^st^ quintile (Ref: 5^th^ quintile)	-	0.040**	0.038**	0.039**	0.040**	0.040**	0.035**	0.029*
		(0.017)	(0.017)	(0.017)	(0.017)	(0.019)	(0.017)	(0.017)
2^nd^ quintile	-	0.040**	0.034**	0.039**	0.040**	0.040**	0.035**	0.030*
		(0.016)	(0.016)	(0.016)	(0.016)	(0.016)	(0.017)	(0.017)
3^rd^ quintile	-	0.030**	0.026*	0.029**	0.030**	0.030**	0.027*	0.023
		(0.014)	(0.014)	(0.014)	(0.014)	(0.015)	(0.015)	(0.015)
4^th^ quintile	-	0.030**	0.026*	0.029**	0.030**	0.030**	0.027*	0.023
		(0.014)	(0.014)	(0.014)	(0.014)	(0.015)	(0.015)	(0.015)
Norms of reciprocity (high)	-	-	-	-	0.004	-	-	0.011
					(0.008)			(0.011)
Norms of association (high)	-	-	-	-	-	−0.001	-	0.011
						(0.008)		(0.011)
*Neighbourhood-level variables*								
Living environment deprivation index	-	−0.001	−0.002	−0.001	−0.001	−0.001	−0.002	−0.002
		(0.005)	(0.005)	(0.005)	(0.005)	(0.005)	(0.005)	(0.005)
% Neighbourhood personalised trust (high)	-	-	−0.073***	-	-	-	-	−0.105***
			(0.026)					(0.030)
% Neighbourhood generalised trust (high)	-	-	-	0.007	-	-	-	0.113**
				(0.038)				(0.044)
% Neighbourhood reciprocity (high)	-	-	-	-	−0.029	-	-	−0.064
					(0.026)			(0.097)
% Neighbourhood associational activity (high)	-	-	-	-	-	−0.003	-	0.042
						(0.025)		(0.038)
% Financial group membership	-	-	-	-	-	-	−0.073**	−0.065**
							(0.033)	(0.033)
% Production group membership	-	-	-	-	-	-	−0.040	−0.041
							(0.151)	(0.150)
% Community service group membership	-	-	-	-	-	-	0.041	0.064
							(0.068)	(0.067)
% Private interest group membership	-	-	-	-	-	-	0.023	0.018
							(0.064)	(0.063)
% Political group membership	-	-	-	-	-	-	−0.023	−0.011
							(0.052)	(0.051)
Var(neighbourhood)	0.0042	0.0038	0.0034	0.0038	0.0038	0.0038	0.0038	0.0034
Var(household)	0.0096	0.0085	0.0083	0.0084	0.0089	0.0085	0.0085	0.0083
Var(residual)	0.0899	0.0869	0.0869	0.0869	0.0869	0.0869	0.0869	0.0868
No. of observations	8866	8866	8866	8866	8866	8866	8866	8866

*Note*: Reference category for race is “White,” and education is “No school or some primary school.” *p ≤ 0.10, **p ≤ 0.05, ***p ≤ 0.01.

The variance components in Table 3 provide information to assess the regression models in terms of *inter alia* the variations at each level. There are minimal changes in the variance components between the null model and the subsequent models in Table 3. Also, the variance at the individual level is larger in comparison to the variance at the household and neighbourhood levels. These indicate that the bulk of the variations in the data occur at the individual level rather than at the higher levels. Thus, the analysis may be conducted without imposing the current hierarchical structure. However, we retain the multilevel model as this has been traditionally used in the literature and it provides a holistic framework to understand the concept of social capital in relation to self-rated health.

## Discussion

This study examined the association between individual-, household- and contextual-level (proxied by neighbourhoods) social capital indicators and self-rated health, while controlling for relevant covariates on all three levels. In particular, the predictor variables used were from the first wave of NIDS in 2008, whereas the outcome of interest, self-rated health, was from the second wave of NIDS in 2010.

The full model’s results indicated that individual personalised trust, contextual personalised trust, and membership in a community service group were associated negatively with poor health. That is, both structural and cognitive components of social capital were associated with self-rated health. Contrary to Model 3, where individual generalised trust was significantly associated with poor health but not contextual generalised trust, contextual generalised trust emerged to be a significant predictor in the full model, but not individual generalised trust. Covariates that remained consistently statistically significant predictors of self-rated health between models were: educational attainment, employment, household income quintile (3^rd^) and household size. The first three are well-established social determinants of health, while the last could be a proxy for social support. The associations between all four of these variables and self-rated rated are similar to what some studies have found [5,24]. While potential health confounders such as smoking and obesity were controlled for, they were not found to be significantly associated with self-rated health. This study also considered contextual-level deprivation. Similar to some studies [5,24], this was not found to be significantly associated with self-rated health. Others, however, have found contextual-level deprivation to be significantly associated with self-rated health [44,45]. The discrepancy could be due to the difference in contexts considered i.e. districts in a city vs Census Enumeration Areas, and/or the varying factors that were included in the indices.

It is difficult to make comparisons of association in regard to social capital with other studies – even in the instance where the same dataset was used – as social capital has been conceptualised differently. For example, in Chola and Alaba’s [24] study, civic participation was a dichotomous variable considered only on the individual level which indicated any group membership, or no membership. In this paper, however, the 18 group memberships were classified according to functions as previous work has shown that different groups may impact on health in varying ways [20,23]. Indeed, in Chola and Alaba’s [24] cross-sectional analysis, they found no association between individual-level civic participation and self-rated health. In this paper, however, individual-level group membership in community service groups was associated inversely with poor self-rated health. No other group memberships were found to be significant in the full model (Model 7). It may be relevant to note that Campbell C, Williams B and Gilgen D [20] found that members of stokvels (a financial group) were more likely to have HIV, but members of sports groups were less likely to have HIV. This suggests that group participation impact differently on health; this may be understood from a social network perspective on social capital which points out that different networks contain different resources that are beneficial to health, depending on the health outcome [46]. Regardless of the health outcome considered, previous studies in South Africa did not account for contextual-level group membership/civic participation [20,23-25]. In this paper, however, although this was taken into account, it was found that contextual-level group membership did not appear to be associated with self-rated health (see Model 7).

In addition, contextual-level social capital in Chola and Alaba’s [24] study was defined by a summative index of four items asked at the household level. Two of the four items were conceived of as reciprocity and associational activity at the household level in this paper, and then aggregated to the neighbourhood level. The other two items – perceived aggression of neighbours and perceived safety of neighbourhood – were not conceived of as social capital in this paper but rather intermediate variables of social capital [47].

It appears that the conceptualised personalised trust and generalised trust, as applied in this paper, have different associations with self-rated health. Therefore, personalised and generalised trust could be an imperfect proxy for bonding and bridging types of social capital. Interestingly, social trust in Chola and Alaba’s [24] study considered only generalised trust. In another study which looked at mental health (assessed by the 10-item Center for Epidemiologic Studies for Depression Scale) and social capital also using data from NIDS Wave 1, only personalised trust was regarded as social trust [25]. In this paper, both items of trust were considered and found that lower individual-level personalised trust was more significantly associated with poorer self-rated health. Similarly, Tomita A and Burns JK [25] found that lower levels of individual personalised trust were associated with higher depression scores; and Fujiwara T and Kawachi I [2] found that personalised trust is associated with better self-rated health. Chola L and Alaba O [24] did not find any association with individual generalised trust and self-rated health, similar to this study. Other studies, however, have found that it was associated with better self-rated health [3,30,48]. Additionally, this paper aggregated individual level of trust to the neighbourhood level, and found that higher levels of contextual-level generalised trust was associated with poorer self-rated health in contrast to another study [29]. The conflicting results regarding the association between generalised trust and health may be due to the differences in the way this variable has been constructed between studies. It further suggests that in-depth qualitative research may be required to understand why generalised trust as conceptualised in this paper was positively associated with poor health status in South Africa.

The results in this paper point to important areas of policy focus that are likely to improve health in South Africa. Some of these include the ‘traditional’ social determinants of health such as education, employment, and socio-economic status and have been covered extensively elsewhere [49,50]. Of particular interest, based on the findings in this paper, are the social capital variables that are significantly associated with self-rated health in South Africa. It is important to note that social capital and the so-called social determinants of health have intrinsic values in themselves apart from the relationship with health and health outcome [49]. Thus, any investment in these determinants is valuable on its own and through some pathways, may translate towards improvements in health and health outcomes. In the context of South Africa, the significant social capital such as community service membership in the form of school committee, water committee, development committee, youth groups, women’s association, and men’s association can be encouraged as a way to improve people’s perceived health status and overall quality of life [51].

One of the main strengths of this paper is the use of two waves of nationally representative panel data. Although it does not enable us to infer causation, and the time period between both waves may be too short, it partly controls for reverse causation as social capital accumulated in 2008 necessarily occurred prior to self-rated health in 2010. This paper also considered civic participation differently compared to many other studies where only participation and/or intensity of participation mattered, but not the function of the groups. It is plausible that different types of social capital can be derived from different groups; and in turn, depending on the health outcome of interest, one may find varying associations between social capital and health. Further, this study considered both cognitive (trust, reciprocity and associational activity) and structural (group membership) aspects of social capital at both the individual and contextual levels. However, there are some limitations. Because a secondary data source was used, it limited the dimensions of social capital that can be considered, namely bonding/bridging/linking social capital. In addition, the outcome, self-rated health, and “exposure” of interest, social capital, are both self-reported. However, this paper is in line with most of the studies that examine a similar relationship as they used self-reported indicators. Also, the degree of involvement in groups could not be determined, which could be a function of the ‘amount’ of social capital one has. Another important variable that could not be considered is whether a person has moved into his/her residence recently. A person new to a neighbourhood is likely to have lower social capital compared to someone who has lived there for longer [52]. Similarly, only contextual-level social capital aggregated from individual-level indicators was used as ecological measures of social capital were not available. Some distinction has been made with regards to integral and derived group- or contextual-level variables [53]. The aggregation done in this paper corresponds to the derived variables, which may not be measuring higher level properties per se [54]. Thus, the inclusion of these derived variables and their individual-level analogue in the models in Table 3 may be similar to over-correction especially when there is little variability in the individual-level data. Another issue is that the neighbourhoods defined in this study, which are based on Census Enumeration Areas, may not be the same spatial areas as those in which the social capital indicators considered have an effect. However, the Census Enumeration Areas have been used in similar analysis and represent the most meaningful unit based on how household listing is done.

## Conclusion

Using two waves of nationally representative longitudinal data, this study has shown that both individual- and contextual-level social capital were significantly associated with self-rated health. It further adds to the current evidence that structural and cognitive social capital contributes independently to self-rated health from a developing country’s perspective. While lower levels of individual-level personalised trust were associated with poorer self-rated health, unsurprisingly, socioeconomic conditions such as educational attainment, employment status and household income were important predictors of self-rated health. Evidence from this study suggests that policy makers may want to consider policies that impact socioeconomic conditions as well as social capital. While some of these policies are linked to the social determinants of health, we contend that the significant social capital including community service membership can be encouraged through policy in a way that is in line with the values of the people. This is likely to impact on health and quality of life generally and lead to a reduction in the burden of disease in South Africa considering the historic context of the country.

## Endnotes

^a^Depending on the part of the country where they operate, stokvels could also be known as “gooi-goois”, “pooling clubs”, “eStokini”, “stokies”, “umgalelos”, “mahodisana”, “mogodisô”, or “kuholisana.” In essence, a stokvel is a voluntary association and a type of “informal credit-rotating association in which a group of people enter into an agreement to contribute a fixed amount of money to a common pool on a weekly or monthly basis or as frequently as the members may agree upon. The contributions or a portion of them are paid out by the association in rotation or in a time of need, depending on the rules of the particular stokvel.” ([36] p.21).

^b^This is equivalent to US$ 20.

## References

[1] Beaudoin CE (2009). Bonding and bridging neighborliness: an individual-level study in the context of health. Soc Sci Med.

[2] Fujiwara T, Kawachi I (2008). Social capital and health: a study of adult twins in the US. Am J Prev Med.

[3] Giordano GN, Björk J, Lindström M (2012). Social capital and self-rated health–a study of temporal (causal) relationships. Soc Sci Med.

[4] Giordano GN, Lindstrom M (2010). The impact of changes in different aspects of social capital and material conditions on self-rated health over time: a longitudinal cohort study. Soc Sci Med.

[5] Han S (2013). Compositional and contextual associations of social capital and self-rated health in Seoul, South Korea: a multilevel analysis of longitudinal evidence. Soc Sci Med.

[6] Mohnen SM, Groenewegen PP, Völker B, Flap H (2011). Neighborhood social capital and individual health. Soc Sci Med.

[7] Woolcock M, Narayan D (2000). Social capital: implications for development theory, research, and policy. World Bank Res Obs.

[8] Szreter S, Woolcock M (2004). Health by association? Social capital, social theory, and the political economy of public health. Int J Epidemiol.

[9] Ferlander S (2007). The importance of different forms of social capital for health. Acta Sociologica.

[10] Bourdieu P, Richardson JG (1986). The forms of capital. Handbook of theory and research for the sociology of education.

[11] Coleman JS (1990). Foundations of social theory.

[12] Putnam RD (1995). Bowling alone: America’s declining social capital. J Democr.

[13] Ayé M, Champagne F, Contandriopoulos AP (2002). Economic role of solidarity and social capital in accessing modern health care services in the Ivory Coast. Soc Sci Med.

[14] Shortt SE (2004). Making sense of social capital, health and policy. Health Policy.

[15] Human Sciences Research Council (2004). Social cohesion and social justice in South Africa.

[16] Ramphele M. Social disintegration in the black community: implications for transformation. Monitor: J Hum Rts Trust. 1991:7–16.

[17] Burns J (2009). Wellbeing and social cohesion: analysis of the NIDS wave 1 dataset, discussion paper #7.

[18] Islam MK, Merlo J, Kawachi I, Lindström M, Gerdtham U-G (2006). Social capital and health: does egalitarianism matter? A literature review. Int J Equity Health.

[19] World Bank (2013). Gini index from World Databank: world development indicators.

[20] Campbell C, Williams B, Gilgen D (2002). Is social capital a useful conceptual tool for exploring community level influences on HIV infection? An exploratory case study from South Africa. AIDS Care.

[21] Cramm JM, Nieboer AP (2011). The influence of social capital and socio-economic conditions on self-rated health among residents of an economically and health-deprived South African township. Int J Equity Health.

[22] Gilbert L, Soskolne V (2003). Self-assessed health—a case study of social differentials in Soweto, South Africa. Health Place.

[23] Pronyk PM, Harpham T, Morison LA, Hargreaves JR, Kim JC, Phetla G (2008). Is social capital associated with HIV risk in rural South Africa?. Soc Sci Med.

[24] Chola L, Alaba O (2013). Association of neighbourhood and individual social capital, neighbourhood economic deprivation and self-rated health in South Africa–a multi-level analysis. PLoS One.

[25] Tomita A, Burns JK (2013). A multilevel analysis of association between neighborhood social capital and depression: evidence from the first South African National Income Dynamics Study. J Affect Disord.

[26] Uphoff N, Dasgupta P, Serageldin I (1999). Understanding social capital: learning from the analysis and experience of participation. Social capital: a multifaceted perspective.

[27] Leibbrandt M, Woolard I, De Villiers L (2009). Methodology: report on NIDS wave 1. Technical paper 1.

[28] Brown M, Daniels R, De Villiers L, Leibbrandt M, Woolard I (2012). National income dynamics study wave 2 user manual.

[29] Kawachi I, Kennedy BP, Glass R (1999). Social capital and self-rated health: a contextual analysis. Am J Public Health.

[30] Kim D, Subramanian S, Kawachi I (2006). Bonding versus bridging social capital and their associations with self rated health: a multilevel analysis of 40 US communities. J Epidemiol Community Health.

[31] Lamarca GA, do C Leal M, Sheiham A, Vettore MV (2013). The association of neighbourhood and individual social capital with consistent self-rated health: a longitudinal study in Brazilian pregnant and postpartum women. BMC Pregnancy Childbirth.

[32] Cronbach LJ (1951). Coefficient alpha and the internal structure of tests. Psychometrika.

[33] Idler EL, Benyamini Y (1997). Self-rated health and mortality: a review of twenty-seven community studies. J Health Soc Behav.

[34] DeSalvo KB, Bloser N, Reynolds K, He J, Muntner P (2006). Mortality prediction with a single general self‐rated health question. J Gen Intern Med.

[35] Ardington C, Gasealahwe B (2012). Health: analysis of the NIDS wave 1 and 2 datasets, working paper #80.

[36] Schulze W (1997). The origin and legal nature of the Stokvel (Part 1). S Afr Merc LJ.

[37] Maluccio J, Haddad L, May J (2000). Social capital and household welfare in South Africa, 1993–98. J Dev Stud.

[38] von Maltitz MJ (2005). The relationship between social capital, welfare and health in South Africa.

[39] Filmer D, Pritchett L (2001). Estimating wealth effects without expenditure data-or tears: an application to educational enrollments in states of India. Demography.

[40] Wright G, Noble M (2009). The South African Index of Multiple Deprivation 2007 at municipality level (technical report).

[41] United Nations Development Programme (2003). South Africa Human Development Report 2003. The challenge of sustainable development in South Africa: unlocking people’s creativity.

[42] O’Donnell O, Van Doorslaer E, Wagstaff A, Lindelow M (2008). Analyzing health equity using household survey data: a guide to techniques and their implementation.

[43] StataCorp (2009). Stata: release 11 - statistical software.

[44] Kavanagh AM, Turrell G, Subramanian S (2006). Does area-based social capital matter for the health of Australians? A multilevel analysis of self-rated health in Tasmania. Int J Epidemiol.

[45] Poortinga W (2012). Community resilience and health: the role of bonding, bridging, and linking aspects of social capital. Health Place.

[46] Van der Gaag M, Webber M, Kawachi I, Subramanian SV, Kim D (2008). Measurement of individual social capital. Social capital and health.

[47] Harpham T, Grant E, Thomas E (2002). Measuring social capital within health surveys: key issues. Health Policy Plan.

[48] Hurtado D, Kawachi I, Sudarsky J (2011). Social capital and self-rated health in Colombia: the good, the bad and the ugly. Soc Sci Med.

[49] Marmot M, Marmot M, Wilkinson R (2005). Introduction. Social determinants of health.

[50] Marmot M, Friel S, Bell R, Houweling T, Taylor S (2008). Closing the gap in a generation: health equity through action on the social determinants of health. Lancet.

[51] García EL, Banegas J, Pérez-Regadera AG, Cabrera RH, Rodríguez-Artalejo F (2005). Social network and health-related quality of life in older adults: a population-based study in Spain. Qual Life Res.

[52] Glaeser EL, Laibson D, Sacerdote B (2002). An economic approach to social capital. Econ J.

[53] Diez-Roux AV (1998). Bringing context back into epidemiology: variables and fallacies in multilevel analysis. Am J Public Health.

[54] Diez RouX AV (2002). A glossary for multilevel analysis. J Epidemiol Community Health.

